# Plant growth-promoting activities of *Streptomyces* spp. in sorghum and rice

**DOI:** 10.1186/2193-1801-2-574

**Published:** 2013-10-29

**Authors:** Subramaniam Gopalakrishnan, Vadlamudi Srinivas, Meesala Sree Vidya, Abhishek Rathore

**Affiliations:** International Crops Research Institute for the Semi-Arid Tropics (ICRISAT), Patancheru, 502 324 Andhra Pradesh India

**Keywords:** Antagonistic *Streptomyces*, Plant growth promotion, Field evaluation, *Fusarium oxysporum* f. sp. *ciceri*, Sorghum, Rice

## Abstract

Five strains of *Streptomyces* (CAI-24, CAI-121, CAI-127, KAI-32 and KAI-90) were earlier reported by us as biological control agents against *Fusarium* wilt of chickpea caused by *Fusarium oxysporum* f. sp. *ciceri* (FOC). In the present study, the *Streptomyces* were characterized for enzymatic activities, physiological traits and further evaluated in greenhouse and field for their plant growth promotion (PGP) of sorghum and rice. All the *Streptomyces* produced lipase, β-1-3-glucanase and chitinase (except CAI-121 and CAI-127), grew in NaCl concentrations of up to 6%, at pH values between 5 and 13 and temperatures between 20 and 40°C and were highly sensitive to Thiram, Benlate, Captan, Benomyl and Radonil at field application level. When the *Streptomyces* were evaluated in the greenhouse on sorghum all the isolates significantly enhanced all the agronomic traits over the control. In the field, on rice, the *Streptomyces* significantly enhanced stover yield (up to 25%; except CAI-24), grain yield (up to 10%), total dry matter (up to 18%; except CAI-24) and root length, volume and dry weight (up to 15%, 36% and 55%, respectively, except CAI-24) over the control. In the rhizosphere soil, the *Streptomyces* significantly enhanced microbial biomass carbon (except CAI-24), nitrogen, dehydrogenase (except CAI-24), total N, available P and organic carbon (up to 41%, 52%, 75%, 122%, 53% and 13%, respectively) over the control. This study demonstrates that the selected *Streptomyces* which were antagonistic to FOC also have PGP properties.

## Introduction

Plant growth-promoting (PGP) microbes are rhizosphere associated organisms that colonize the rhizosphere and rhizoplane and improve plant growth when artificially inoculated onto the seeds or into soil. PGP microbes may promote plant growth either by direct stimulation such as iron chelation, phosphate solubilization, nitrogen fixation and phytohormone production or by indirect stimulation such as suppression of plant pathogens and induction of resistance in host plants against pathogens (Basak and Biswas 2009; Hao et al. 2011; Panhwar et al. 2012). The opportunities of PGP microbes include alternating applications of PGP microbes as bio-fungicides with inorganic fungicides to manage fungicide resistance and to reduce the number of fungicide applications per year. PGP microbes also plays an important role in integrated nutrient management programs with a goal of reducing run-off of unused fertilizers and the environment damage that results (Kloepper 2009; Gopalakrishnan et al. 2013a).

PGP and biological control of plant pathogens has been addressed using actinomycete, bacterial and fungal antagonists. For example, strains of *Streptomyces*, *Bacillus*, *Pseudomonas* and *Trichoderma* were effective not only in helping plants to mobilize and acquire nutrients but also to control plant pathogens (Postma et al. 2003; Khan et al. 2004; Perner et al. 2006; Borriss et al. Borriss et al. 2011; Gopalakrishnan et al. 2011a, b). Microorganisms with PGP and biocontrol potential were found at high incidence in compost, forest and pasture soils (Torsvik et al. 2002; Tinatin and Nurzat 2006). PGP traits of actinomycetes have been reported on pea (Tokala et al. 2002), bean (Nassar et al. 2003), tomato (El-Tarabily 2008), wheat (Sadeghi et al. 2012) and rice (Gopalakrishnan et al. 2012a).

Five strains of *Streptomyces* spp. (CAI-24, CAI-121, CAI-127, KAI-32 and KAI-90), isolated from herbal vermi-compost, were earlier reported by us as having potential for biocontrol of *Fusarium* wilt in chickpea, caused by *Fusarium oxysporum* f. sp. *ciceri* (FOC; Gopalakrishnan et al. 2011b). Also, the selected *Streptomyces* strains were reported to produce siderophore, indole acetic acid (IAA; except KAI-90), hydrocyanic acid, cellulase (only KAI-32 and KAI-90) and protease (only for CAI-24 and CAI-127). The objective of this study was to further characterize the five *Streptomyces* strains for their enzymatic activities (chitinase, lipase and β-1-3-glucanase), physiological traits (salinity, pH, temperature, fungicide tolerance and antibiotic resistance) and to evaluate for their PGP traits under greenhouse and field conditions in sorghum and rice.

## Materials and methods

### *Streptomyces* strains

Five strains of *Streptomyces*, isolated from herbal vermi-compost, CAI-24 (*Streptomyces tsusimaensis*; NCBI Accession Number: JN400112), CAI-121 (*Streptomyces caviscabies*; NCBI Accession Number: JN400113), CAI-127 (*Streptomyces setonii*; NCBI Accession Number: JN400114), KAI-32 (*Streptomyces africanus*; NCBI Accession Number: JN400115) and KAI-90 (*Streptomyces* spp.; NCBI Accession Number: JN400116), reported earlier by us as potential for biocontrol traits against *Fusarium* wilt in chickpea (Gopalakrishnan et al. 2011b), were further studied in this investigation.

### Evaluation of *Streptomyces* strains for their enzymatic activities

#### Chitinase, lipase and β-1,3-glucanase production

Chitinase and lipase production was conducted for all five strains (CAI-24, CAI-121, CAI-127, KAI-32 and KAI-90) of *Streptomyces* as described by Hirano and Nagao (1988) and Bhattacharya et al. (2009), respectively. The *Streptomyces* were streaked onto chitin agar (for chitinase) and Tween 80 agar (for lipase) and the Petri dishes were incubated at 28 ± 2°C for five days. At the end of incubation, the Petri dishes were observed for haloes around the colonies, indicating the production of these enzymes. All the treatments were replicated three times and the experiment was conducted three times. Chitinase production was recorded on a 0–5 rating scale as follows: 0 = no halo; 1 = halo of 1–5 mm; 2 = halo of 6–10 mm; 3 = halo of 11–15 mm; 4 = halo of 16–20 mm and 5 = halo of 21 mm and above. Observations for lipase production were recorded on a 0–5 rating scale as follows: 0 = no halo; 1 = positive; 2 = halo of 1–3 mm; 3 = halo of 4–6 mm; 4 = halo of 7–10 mm and 5 = halo of 11 mm and above.

β-1,3-glucanase was measured as per the protocols of Singh et al. (1999). All five *Streptomyces* strains were cultured individually in Tryptic soy broth, supplemented with 1% (weight/volume) colloidal chitin, at 28°C for four days. At the end of the incubation, the cultures were processed as per Gopalakrishnan et al. (2013b). The development of a dark red color indicated the presence of β-1,3-glucanase. Treatments were replicated three times and the experiment was conducted three times. One unit of β-1,3-glucanase activity was defined as the amount of enzyme that liberated 1 μmol of glucose hour^-1^ under defined conditions.

### Evaluation of *Streptomyces* strains for their physiological traits

#### Salinity, pH and temperature

The five *Streptomyces* were streaked on Bennett’s agar with NaCl concentrations ranging from 0 − 8% (at intervals of 2%) and pH ranging from 5–13 (at intervals of 2 pH units); for pH 3, Bennett’s broth was used and the intensity of growth was measured at 600 nm in a spectrophotometer after incubation at 28 ± 2°C for five days. For evaluating the effect of temperature, the five *Streptomyces* strains were streaked on Bennett’s agar and incubated at 20, 30 and 40°C for five days, but for 50°C, Bennett’s broth was used and the intensity of growth was measured at 600 nm in a spectrophotometer.

#### Fungicide tolerance

Fungicide tolerance studies were conducted for Thiram (dimethylcarbamothioylsulfanyl *N*, *N*-dimethylcarbamodithioate), Bavistin (carbindozim 50%; methyl benzimidazol-2-ylcarbamate), Benlate (benomyl 50%; methyl [1-[(butylamino) carbonyl]-1H-benzimidazol-2-yl] carbamate), Captan (captan 50%; *N*-trichloromethylthio-4-cyclohexene-1, 2-dicarboximide), Benomyl (methyl [1-[(butylamino)carbonyl]-1H-benzimidazol-2-yl]carbamate) and Radonil (*N*-(2,6-dimethylphenyl)-*N*-(methoxyacetyl) alanine methyl ester) at field application levels of 3000, 2500, 4000, 3000, 3000 and 3000 ppm concentrations, respectively. The required quantities of fungicides were dissolved in sterilized Milli Q water and mixed with Bennett’s agar just before pouring into Petri plates. Upon solidification, the five *Streptomyces* were inoculated and incubated at 28 ± 2°C for five days.

All the physiological traits were replicated three times and the experiment was conducted three times. Responses of the five *Streptomyces* to salinity, pH, temperature and fungicide tolerance were recorded as follows: 0 = no growth; 1 = slight growth; 2 = moderate growth and 3 = good growth.

### Evaluation of *Streptomyces* strains for their PGP potential under greenhouse conditions on sorghum

The five *Streptomyces* strains antagonistic against FOC (CAI-24, CAI-121, CAI-127, KAI-32 and KAI-90) were evaluated in a greenhouse for their PGP potential on sorghum. A total of six treatments (five *Streptomyces* strains + un-inoculated control) were made with six replications. Pot mixture (1000 g) was prepared by mixing red soil, sand and farmyard manure at 3:2:2 and placed in 20 cm diam plastic pots. Sorghum seeds (variety ICSV 112) were surface sterilized with sodium hypochlorite (2.5% for 5 min) and rinsed with sterilized water (8 times) before being transferred into the respective test *Streptomyces* strains (10^8^ cfu ml^−1^; grown in starch casein broth (SCB) separately) for an hour before being sown in the pots (six seeds/pot but thinned to three after one week). Booster doses of *Streptomyces* strains (5 ml per seedling, 10^8^ cfu ml^−1^) were applied at 15, 30 and 45 days after sowing by the soil drench method. Growth parameters including plant height, leaf area, stem and leaf dry weight, root length, root surface area, root volume and root dry weight were determined at day 60 after sowing.

### Evaluation of *Streptomyces* strains for their PGP potential under field conditions on rice

The field trial was performed in the 2011 Kharif (rainy) season at ICRISAT, Patancheru, Hyderabad, India. The details of the experimental site, soil and the experiment were described previously (Gopalakrishnan et al. 2012a). The experiment was conducted in a randomized complete block design with three replicates and subplot sizes of 10 × 7.5 m. Rice was grown by the system of rice intensification (SRI) method as described by Uphoff (2003). The five *Streptomyces* (CAI-24, CAI-121, CAI-127, KAI-32 and KAI-90) were grown on SCB at 28°C for five days before evaluated for their PGP traits. The control plots contained no *Streptomyces*. The 11-day-old single seedlings were uprooted from the nursery (laid next to the experimental field), their roots dipped in the respective *Streptomyces* broth (containing 10^7^ cfu mL^−1^) for 60 min and transplanted on 4th August 2011 at a spacing of 25 × 25 cm (row-to-row and plant-to-plant spacing). Plants were inoculated with respective *Streptomyces* strains (1500 ml; 10^7^ cfu ml^−1^) once in every two weeks until the flowering stage along with the irrigation water. Irrigation management was performed as recommended for the SRI method (alternate wetting and drying method; Uphoff et al. 2009). Weeding was performed by a Cono-weeder at intervals of 15 days after transplanting until the flowering stage. The crop was not sprayed with any chemicals as no serious insect-pests/pathogens were noticed. The recommended dose of N:P:K (120: 60:40 kg ha^−1^) were applied through compost and farm yard manure. The crop was harvested manually on 23rd November 2011 and observed for plant height, number of tillers, primary and secondary panicle numbers, panicle length, stover yield, grain yield, total dry matter and 1000 seed weight. Root samples were collected from 0–15 cm and 15–30 cm soil profile after harvesting and analyzed for root length density (EPSON expression 1640×, Japan), volume and dry weight (dried in an oven at 60°C for 72 h). Soil samples (from 0–15 cm soil profile) were collected at harvesting and analyzed for soil chemistry (% organic carbon, available phosphorous and total nitrogen as per the standardized protocols of Nelson and Sommers (1982); Olsen and Sommers (1982) and Novozamsky et al. (1983), respectively) and biological analysis (dehydrogenase activity, microbial biomass nitrogen and microbial biomass carbon as per Casida (1977), Brooks et al. (1985) and Anderson and Domsch (1989), respectively).

### Statistical analysis

The greenhouse and field experiment data were subjected to ANOVA (GenStat 10.1 version 2007, Lawes Agricultural Trust, Rothamsted Experimental Station) to evaluate the efficiency of the PGP agents. Significance of differences between the treatment means were tested at *P* = 0.01 and 0.05.

## Results

### Evaluation of *Streptomyces* strains for their enzymatic activities and physiological traits

**Figure 1:**
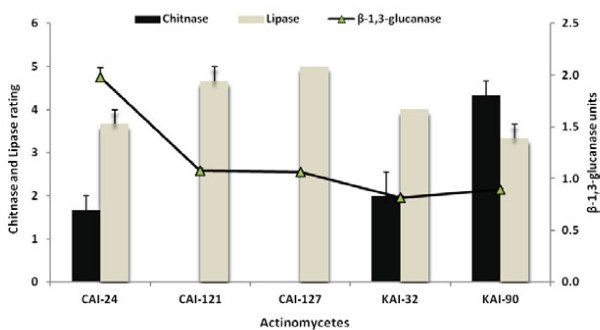
**Evaluation of the five** ***Streptomyces*** **strains antagonistic to FOC for their enzymatic activities.**

**Figure 2:**
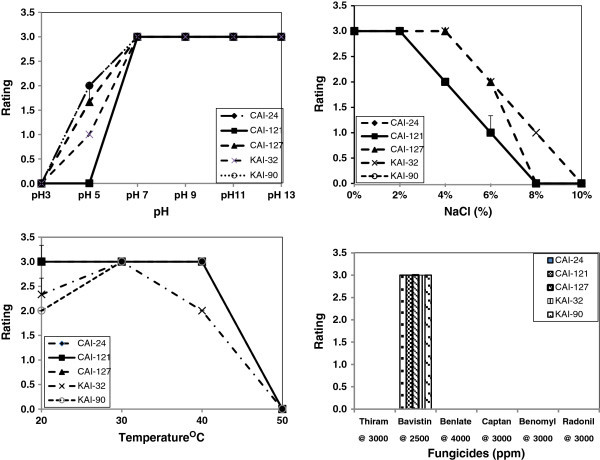
**Evaluation of the five** ***Streptomyces*** **strains antagonistic to FOC for their tolerance of salinity, pH, temperature and fungicides (at field level application).**

All five *Streptomyces* strains produced β-1,3-glucanase and lipase but only three strains (CAI-24, KAI-32 and KAI-90) produced chitinase (Figure 1). All strains were able to grow in NaCl up to 6%, at pH values between 5 and 13 and temperatures between 20 and 40°C. They were highly tolerant to Bavistin but sensitive to Thiram, Benlate, Captan, Benomyl and Radonil at field application levels. A pH of 5 and 10% NaCl were discriminatory for the strains, strain CAI-121 showing no growth at pH 5 while others exhibited medium growth whereas strain CAI-127 showed medium growth at 10% NaCl while others exhibited none (Figure 2).

### Evaluation of *Streptomyces* strains for their PGP potential on sorghum under greenhouse conditions

The *Streptomyces* strains significantly enhanced all PGP parameters; plant height (16 − 51%), leaf area (11 − 24%, except KAI-32), stem weight (7 − 39%, except CAI-127), leaf weight (2 − 12%), root length (3 − 18%), root surface area (2 − 11%, except CAI-121), root volume (2 − 19%) and root dry weight (4 − 25%) over the control (Table 1).

**Table 1:** **Evaluation of the five** ***Streptomyces*** **strains antagonistic to FOC for their plant growth promotion potential in sorghum under greenhouse conditions**

Treatment	Plant height (cm)	Leaf area (cm^2^)	Stem weight (g)	Leaf weight (g)	Root length (m plant^-1^)	Surface area (cm^2^plant^-1^)	Root volume (cm^3^plant^-1^)	Root dry weight (g plant^-1^)
CAI-24	114	560	5.42	2.28	90	1003	10.2	0.92
CAI-121	122	580	5.55	2.32	80	905	9.0	0.84
CAI-127	100	624	5.09	2.35	88	922	9.3	0.87
KAI-32	124	507	6.39	2.21	82	991	10.5	0.89
KAI-90	130	589	7.03	2.41	78	973	10.5	1.01
Control	86	504	5.06	2.16	76	903	8.8	0.81
Mean	113	561	5.76	2.29	82	949	9.7	0.89
SE±	4.9***	27.5*	0.471*	0.048*	3.4*	13.9***	0.42*	0.036*
LSD (5%)	14.9	83.0	1.421	0.146	10.0	42.0	1.28	0.109
CV%	9	10	16	4	8	3	9	8

SE = standard error; LSD = least significant difference; CV = coefficient of variance; * = statistically significant at p = 0.05; *** = statistically significant at p = 0.001.

### Evaluation of *Streptomyces* strains for their PGP potential under field conditions on rice

Under field conditions in rice, the five *Streptomyces* strains significantly enhanced plant height (cm plant^-1^), tillers (m^-2^, 9 − 28%), primary and secondary panicle number (plant^-1^), panicle length (cm), stover and grain yield (g m^-2^, 6 − 25% and 0.2 − 10%, respectively), total dry matter (g m^-2^, 3 − 18%) and test seed weight (g) over the control (Table 2). Root length (mm^-2^, 3 − 15%), root volume (cm^-3^ m^-2^, 1 − 35%) and root dry weight (gm^-2^, 2 − 55%) were also found significantly enhanced in both the soil depths (0–15 cm and 15–30 cm) in all the *Streptomyces* strains (except CAI-24) inoculated plots over the control (Table 3). The soil biological activities (microbial biomass carbon [μg g^-1^ soil, 0.5 − 41%], microbial biomass nitrogen [μg g^-1^ soil, 7 − 52%] and dehydrogenase activity [μg TPF g^-1^ soil 24 h^-1^, 2 − 75%]) in the top 15 cm rhizosphere soils were significantly higher in *Streptomyces* inoculated treatments (except CAI-24) at harvest, over the control (Table 4). The total N, available P and organic carbon% (chemical activities) were also found significantly enhanced in the top 15 cm of rhizosphere soils of *Streptomyces* treated plants (by 67 − 122%, 32 − 53% and 0 − 13%, respectively) at harvesting than those of the controls (Table 4).

**Table 2:** **Effect of FOC antagonistic** ***Streptomyces*** **on the morphology and yield potential of rice cultivation**

Treatment	Plant height (cm)	Tillers number (m^-2^)	Primary panicles number	Secondary panicles number	Panicle length (cm)	Stover yield (g m^-2^)	Grain yield (g m^-2^)	Total dry matter (g m^-2^)	Test seed weight (g)
CAI-24	85	586	11.9	29.8	24.8	584	587	1170	18.9
CAI-121	81	506	11.8	26.5	24.5	637	583	1221	18.9
CAI-127	80	501	12.1	31.0	24.6	754	619	1373	18.6
KAI-32	82	532	12.2	28.3	25.0	754	640	1394	19.3
KAI-90	81	589	11.7	27.8	24.5	693	587	1279	18.9
Control	80	459	11.4	25.0	24.4	601	582	1183	18.6
Mean	81	529	11.8	28.0	24.6	671	600	1270	18.9
SE ±	0.68***	15.6***	0.06**	0.29***	0.09*	27.8**	9.7**	31.9***	0.03***
LSD (5%)	2.0	47.1	0.22	1.06	0.33	87.7	30.5	100.4	0.11
CV%	2	7	1	2	1	7	3	4	1

SE = standard error; LSD = least significant difference; CV = coefficient of variance; * = statistically significant at p = 0.05; ** = statistically significant at p = 0.01; *** = statistically significant at p = 0.001.

**Table 3:** **Effect of FOC antagonistic** ***Streptomyces*** **on the root development of rice at harvesting stage of rice cultivation**

Treatment	Root length (m m^-2^)			Root volume (cm^-3^m^-2^)			Root dry weight (g m^-2^)		
	0 − 15 cm	15 − 30 cm	Mean	0 − 15 cm	15 − 30 cm	Mean	0 − 15 cm	15 − 30 cm	Mean
CAI-24	3798	375	2087	745	46	396	58.3	2.8	30.5
CAI-121	5859	667	3263	945	81	513	66.7	5.3	36.0
CAI-127	6348	591	3470	1303	81	692	93.5	5.4	49.5
KAI-32	6490	813	3652	1170	84	627	101.9	6.8	54.3
KAI-90	5865	1319	3592	1009	153	581	79.7	9.8	44.8
Control	5776	588	3182	932	81	507	65.0	5.1	35.1
SE ±		121.9 (128.0)***	81.6***		22.5 (21.3)***	16.8***		2.59 (2.51)***	1.89***
Mean	5689	726		1017	88		77.5	5.9	
SE ±		52.3***			8.7***			1.02***	
CV%		7			7			10	

SE = standard error; CV = coefficient of variance; *** = statistically significant at p = 0.001.

**Table 4:** **Effect of FOC antagonistic** ***Streptomyces*** **on soil biological activities and mineral nutrients at harvesting stage of rice cultivation**

Treatment	Microbial biomass C (μg g^-1^soil)	Microbial biomass N (μg g^-1^soil)	Dehydrogenase activity (μg TPF g^-1^soil 24 h^-1^)	Total N (g kg^-1^soil)	Available P (mg g^-1^soil)	Organic carbon (%)
CAI-24	1715	60	94	2.456	0.133	1.49
CAI-121	3293	65	113	1.992	0.117	1.47
CAI-127	2875	88	194	2.644	0.115	1.52
KAI-32	4020	65	135	2.142	0.122	1.66
KAI-90	2946	62	136	2.160	0.129	1.62
Control	2861	58	111	1.190	0.087	1.47
Mean	2952	66	131	2.231	0.117	1.53
SE ±	150.5***	3.7**	9.3***	0.063*	0.005*	0,032*
LSD (5%)	520.7	12.0	29.7	0.229	0.019	0.116
CV%	9	10	12	4	6	3

C = carbon; N = nitrogen; P = phosphorous; SE = standard error; LSD = least significant difference; CV = coefficient of variance; * = statistically significant at 0.05; ** = statistically significant at p = 0.01; *** = statistically significant at p = 0.001.

## Discussion

The five *Streptomyces* strains (CAI-24, CAI-121, CAI-127, KAI-32 and KAI-90) used in the present investigation were earlier reported as not only having potential for biocontrol of *Fusarium* wilt disease in chickpea but also having PGP traits such as IAA (except KAI-90) and siderophore production (Gopalakrishnan et al. 2011b). Microbes producing IAA stimulate plant growth while siderophore producers bind Fe^3+^ from the environment and make it available for plants and their growth (Patten and Glick 2002; Tokala et al. 2002; Hayat et al. 2010). In the present investigation, the five FOC antagonistic *Streptomyces* were further characterized for enzymatic activities, physiological traits and PGP potential on sorghum and rice under greenhouse and field conditions.

In the enzymatic activity studies, all the five strains produced β-1,3-glucanase and lipase but only CAI-24, KAI-32 and KAI-90 produced chitinase. The cell walls of plant pathogens contain β-1,3-glucan, chitin and lipid, which are essential for the pathogen for disease transmission and pathogenesis, and lysis of these by β-1,3-glucanase/chitinase/lipase-producing microbe leads to leakage of cell contents and collapse of the pathogenic fungi (Singh et al. 1999; Lynd et al. 2002; Macagnan et al. 2008). Hence, microorganisms having these traits can be exploited for biological control of plant pathogens, which indirectly promotes the plants. In the physiological traits studies, the five *Streptomyces* strains were able to grow in NaCl up to 6% and at pH values between 5 and 13 and temperatures between 20 and 40°C. The ability of *Streptomyces* spp. to grow in harsh pH and temperature and higher concentration of salinity is reported (Sadeghi et al. 2012). In the present study, all the *Streptomyces* strains were highly tolerant to the fungicide Bavistin at field application level. Therefore, it is concluded that these strains may not only have the capability to survive in harsh environments but are also compatible with the Bavistin and hence can be used in integrated disease management programs.

In the present study, under greenhouse conditions in sorghum and field conditions in rice, the *Streptomyces* strains significantly enhanced all the agronomic observations including root length, volume and dry weight and yield parameters over the un-inoculated control. The *Streptomyces* strains are reported widely in the literature for its PGP potential (Nassar et al. 2003; El-Tarabily 2008; Gopalakrishnan et al. 2012a; 2013b). As hypothesized earlier, the mechanism by which the *Streptomyces* enhanced the morphological and yield parameters on both sorghum and rice could be their PGP traits including IAA and siderophore production (direct stimulation of PGP; Gopalakrishnan et al. 2011b) and or chitinase, lipase and β-1,3-glucanase production (indirect stimulation of PGP; as reported in the present investigation). The effect of soil microbes on PGP including root development has been reported by Birkhofer et al. (2008) and Uphoff et al. (2009).

In the SRI method of rice cultivation, irrigation was done by alternate wetting and drying method. Such a system of irrigation favors soil microbial and biological activities and enhances the availability of mineral nutrients compared to rice grown by flooded cultivation methods (Uphoff et al. 2009; Gopalakrishnan et al. 2012b). In the present investigation, soil biological activities including microbial biomass carbon, nitrogen and dehydrogenase and mineral nutrients including available P, total N and % organic carbon were found to be higher in the five *Streptomyces* inoculated plots over the un-inoculated control plots. Similar results were found in SRI rhizospheres compared with those of the same variety of rice plants grown conventionally (Gayathry 2002). Hence, it can be concluded that the five *Streptomyces* strains survived in the rice rhizosphere and enhanced the soil health conditions. In the present investigation, although roots were not checked for colonization, the data on agronomical (including roots), yield, biological and mineral nutrition studies indicate that the five *Streptomyces* strains had multiplied and colonized the rice roots. Hence, it can be concluded that the five *Streptomyces* strains used in this study were apparently well adapted to the rice and sorghum rhizosphere environments and enhanced the soil health and plant growth conditions. Significant increases in growth and yield of agronomically important crops in response to inoculation with PGP microbes have been repeatedly reported (Biswas et al. 2000; Asghar et al. 2002; Silva et al. 2006). PGP microbes are thought to stimulate plant growth by two mechanisms: (1) by altering hormone balance in the host plant and increasing mineral nutrient solubilization (direct effects); (2) by antagonism towards plant pathogens (indirect effects; Glick 1995). In the present investigation, the five *Streptomyces* strains were found to have both mechanisms, having PGP traits in rice and sorghum (direct effects) and biocontrol traits, against *Fusarium* wilt, in chickpea (indirect effects; Gopalakrishnan et al. 2011b). Broad spectrum PGP and biocontrol agents offer potentially effective novel strategies for controlling multiple pathogens and insect pests. A few of the available biocontrol agents, mostly belonging to *Pseudomonas* spp., have shown broad spectrum antifungal activity (Hass and Keel 2003; Viji et al. 2003; Kishore et al. 2005). Actinomycetes and other bacteria are also reported in the literature as broad spectrum PGP and biocontrol agents for soil-borne fungal plant pathogens and insect-pests, such as *Helicoverpa armigera* and *Spodoptera litura* (El-Tarabily and Sivasithamparam 2006; Sadeghi et al. 2012).

The five *Streptomyces* used in this study were well adapted to not only in the chickpea rhizosphere but also in the sorghum and rice rhizosphere where they promoted plant growth. Hence, these isolates could be used as PGP agents in addition to the biocontrol of *Fusarium* wilt. Though, all the five strains have been demonstrated for their PGP potential in rice KAI-32 and KAI-90 were found to have superiority over other isolates in terms of crop productivity and root development. The broad range of PGP and antifungal activities of the five *Streptomyces* strains demonstrates multiple mechanisms of actions including antibiosis, production of cell wall degrading enzymes and plant growth-promoting hormone (IAA). Therefore, these *Streptomyces* can be considered for isolation of novel secondary metabolites which may be of importance for various biocontrol and PGP applications. Use of biocontrol agents such as these broad-spectrum *Streptomyces* will probably be one of the important tactics for plant disease management in the near future as they allow the reduced use of pesticides and fertilizers that are potential pollutants of the environment.
